# Valve‐in‐valve transcatheter aortic valve replacement to treat multijet paravalvular regurgitation: A case series and review

**DOI:** 10.1002/clc.23504

**Published:** 2020-11-20

**Authors:** Morgan H. Randall, Thomas J. Lewandowski, Calvin Choi, Thomas M. Beaver

**Affiliations:** ^1^ Department of Medicine, Division of Cardiovascular Medicine University of Florida Gainesville Florida USA; ^2^ North Florida/South Georgia Veterans Health System Medical Service, Cardiology Section Gainesville Gainesville Florida USA; ^3^ Department of Surgery, Division of Thoracic and Cardiovascular Surgery University of Florida Gainesville Florida USA

**Keywords:** multijet paravalvular regurgitation, paravalvular regurgitation, transcatheter aortic valve replacement, valve‐in‐valve

## Abstract

Treatment advances for severe symptomatic aortic stenosis including transcatheter and open surgical valve replacement have improved patient survival, length of stay, and speed to recovery. However, paravalvular regurgitation (PVR) is occasionally seen and when moderate or greater in severity is associated with an at least 2‐fold increase in 1 year mortality. While several treatment approaches focused on single‐jet PVR have been described in the literature, few reports describe multijet PVR. Multijet PVR can successfully be treated with a variety of catheter‐based options including valve‐in‐valve (ViV) transcatheter aortic valve replacement (TAVR). We present two patients with at least moderate PVR following aortic valve replacement who were successfully treated with ViV TAVR along with a review of literature highlighting our rationale for utilizing each management approach. Multijet PVR can be treated successfully with ViV TAVR, but additional options such as self‐expanding occluder devices and bioprosthetic valve fracture have a role as adjunctive treatments to achieve optimal results. The etiology of multijet PVR can differ between patients, this heterogeneity underscores the paucity of data to guide treatment strategies. Therefore, successful treatment of multijet PVR requires familiarity with available therapeutic options to achieve optimal results and, by extension, decrease patient mortality.

**Abbreviations**
PVR: paravalvular regurgitation
TAVR: transcatheter aortic valve replacement
TEE: transesophageal echocardiogram
ViV: valve‐in‐valve

## 1 BACKGROUND

Bioprosthetic valve implantation has become a mainstay of management in patients with symptomatic aortic stenosis with both surgical and transcatheter methods demonstrating survival benefit.^1^, ^2^ However, a number of potential procedural complications exist including paravalvular regurgitation (PVR).^3^ Although PVR has decreased with improved valve design and deployment techniques, when moderate or severe it is associated with a 2‐fold or greater 1 year mortality.^3^, ^4^

Strategies to address PVR are individualized for each patient with potential options including redo surgical valve replacement, transcatheter self‐expanding occluder devices, balloon valvuloplasty, or valve‐in‐valve (ViV) transcatheter aortic valve replacement (TAVR). While literature exists regarding the management of PVR, there is a paucity of evidence in patients with multiple jet PVR. Therefore, we present two patients along with a review of relevant literature upon to inform treatment decision‐making.

## 2 CASE 1

An 87‐year‐old male presented 9 months post TAVR at another institution with PVR complaining of NYHA class III symptoms of increasing fatigue, dyspnea on exertion, orthopnea and paroxysmal nocturnal dyspnea. A 26 mm Edwards Sapien 3 bioprosthetic TAVR valve had been implanted, but the intraoperative sizing at that time was based on transesophageal echocardiography (TEE) instead of CT scan imaging. Unfortunately, that procedure was also complicated by a Stanford Type B descending thoracic aortic dissection that required emergent thoracic stent coverage to treat visceral malperfusion. The patient developed mesenteric ischemia requiring bowel resection. After a prolonged hospitalization, he was found to have moderate aortic regurgitation on follow‐up echocardiography, which was managed medically until his presentation to our center.

Physical exam revealed a diastolic murmur and 1+ lower extremity edema. A TEE revealed moderate paravalvular aortic insufficiency comprised of two jets: the largest was along the postero‐medial aspect including approximately 20% of the valve circumference, while a smaller jet was along the antero‐lateral aspect of the valve and involved an additional 10% of the valve circumference. TEE also showed dilation of the mitral annulus with subsequent moderate mitral regurgitation and reduced left ventricular ejection fraction of 40% to 45%.

Given his frailty, comorbidities and history of aortic dissection he was not an open surgical candidate. Transcatheter methods including Amplatz plug versus redo‐TAVR were considered and the patient underwent computed tomography evaluation of the aortic root and review of his previous left heart catheterization.

Given the multiple jet PVR consensus formed that ViV TAVR would be a superior solution to surgical or transcatheter placement of vascular occluder devices. Based on repeat computed tomography and transesophageal imaging we hypothesized the initial valve was undersized, resulting in the multiple foci of PVR. We evaluated for nodular calcification but in our experience, this leads to a focal paravalvular leak adjacent to the focus of calcification rather than multiple jets and thus was thought to be less likely. The team considered further expansion of the previously implanted valve, but mechanical damage to the valve was felt to be a potential complication that could result in detrimental results. Our intended strategy, therefore, became to place a new valve in a more ventricular position and take advantage of the inflow skirt to potentially seal all foci of PVR.

**FIGURE 1:**
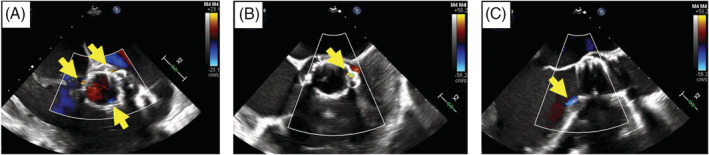
Intraoperative TEE images of patient 1. A, Multiple jets of PVR (yellow arrows; Supplemental Video 1). B, PVR after ViV implantation (yellow arrow; Supplemental Video 2). C, Post‐procedure trivial PVR (yellow arrow; Supplemental Video 3). PVR, paravalvular regurgitation; TEE, transesophageal echocardiogram; ViV, valve‐in‐valve

Standard approach for redo‐TAVR was performed with additional precautions for severe vascular disease and the presence of a Thoracic Stent Graft in the descending aorta such as careful wire manipulation and catheter exchange. Intraoperative TEE revealed three jets of paravalvular leak with severe aortic insufficiency (Figure 1A).

A 26 mm Edwards Sapien 3 ViV was deployed under rapid ventricular pacing over a Confida wire (Medtronic, Minneapolis, Minnesota) slightly lower than the prior valve with improvement in AI. This size was chosen due to concern that a larger 29 mm valve may incompletely expand which would result in less ideal hemodynamic parameters and thus be ineffective. Post‐implantation balloon aortic valvuloplasty was performed with an additional 3 mL above the nominal inflation volume of 23 mL. This maneuver flared the lower end of the Sapien 3 valve to take advantage of the outer skirt on its ventricular end and inhibit PVR. Intraoperative TEE subsequently revealed only mild residual PVR from the left coronary sinus (Figure 1B). As this patient presented with debilitating heart failure symptoms and because of our institutional experience and comfort with addressing such defects, the decision was made to further optimize the result in this case. This defect was therefore crossed with a flexible soft tipped straight 0.035″ wire (Terumo, Somerset, New Jersey) followed by a CXI catheter (Cook Medical, Bloomington, Indiana) into the left ventricle. An Amplatzer Duct Occluder II (Abbott, Abbott Park, Illinois) was sized for the device waist to be 1.5 to 2 times the measured PVR jet width. This occluder device was then deployed under TEE and fluoroscopic guidance with final TEE revealing only trivial aortic regurgitation (Figure 1C). The patient was discharged on postoperative day 2 with immediate improvement to NYHA Class I symptoms.

## 3 CASE 2

A 69‐year‐old male presented for evaluation of known moderate to severe PVR with symptoms of progressive dyspnea on exertion, orthopnea, and weight gain 2 months after coronary artery bypass surgery and aortic valve replacement at another institution. His past medical history included nonsmall cell lung cancer, type 2 diabetes mellitus, chronic obstructive pulmonary disease, and 3‐vessel coronary artery disease. The patient had originally presented after a non‐ST elevation myocardial infarction with severe aortic stenosis and severe coronary artery disease. At the time of his 3‐vessel coronary artery bypass grafting and open surgical aortic valve replacement the surgery was complicated by inability to advance a 23 mm sizer through the sinotubular junction due to severe calcium, which resulted in the placement of an undersized 21 mm Magna valve (Edwards, Irvine, California). At the completion of the surgery, intraoperative imaging revealed moderate to severe aortic regurgitation, but the calcium was felt prohibitive for repair and the patient was referred to our institution.

On presentation, the patient was wheelchair bound with New York Heart Association Class IV symptoms and was hypoxic requiring supplemental oxygen. Examination identified a diastolic murmur, 4 + lower extremity edema, and bibasilar rales.

**FIGURE 2:**
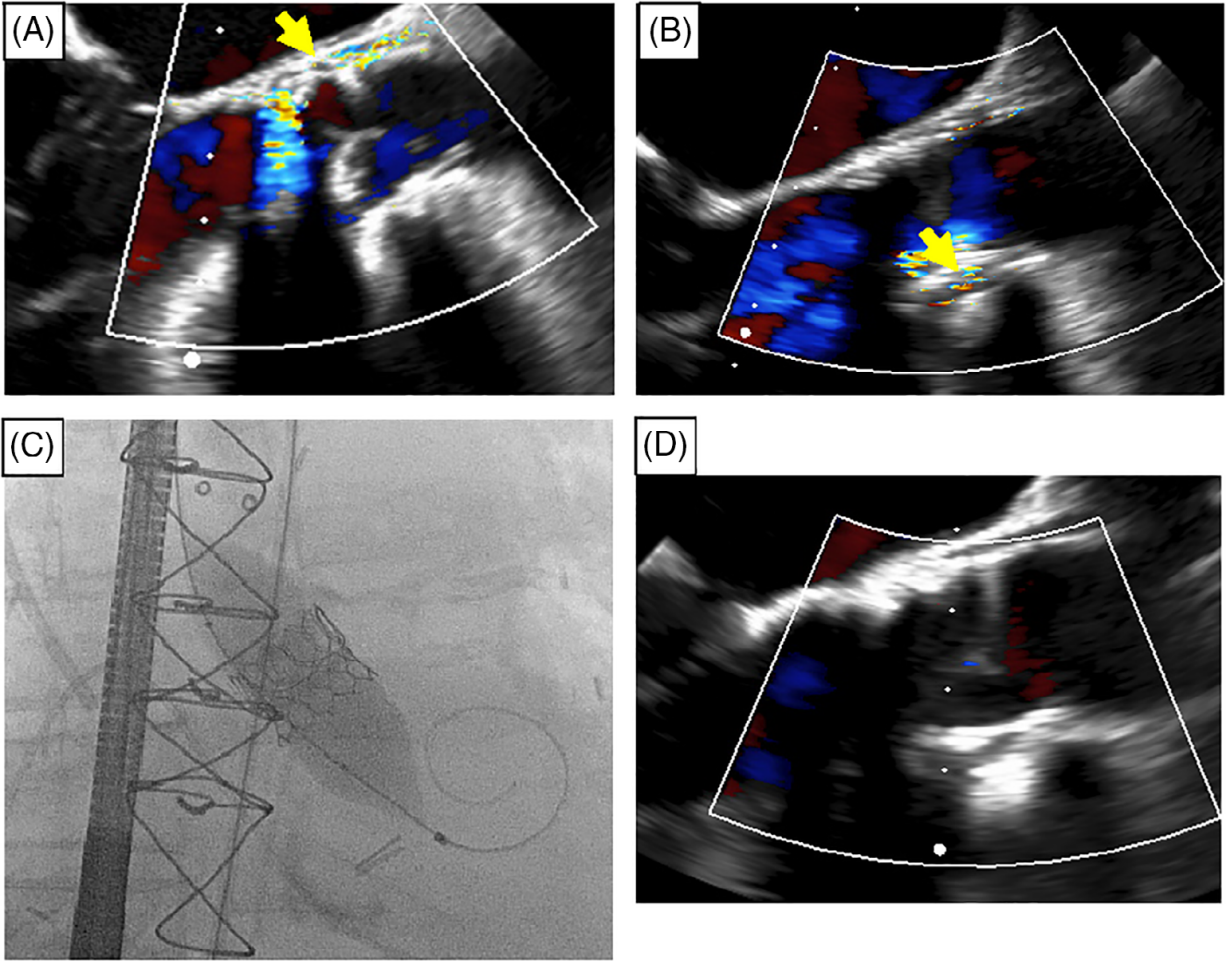
Intraoperative images of patient 2. A, TEE image of first PVR jet prior to procedure (yellow arrow; Supplemental Video 4). B, TEE image of second PVR prior to procedure (yellow arrow; Supplemental Video 5). C, Cine angiography of balloon post‐dilation (Supplemental Video 6). D, TEE image after completion of the procedure (Supplemental Video 7). PVR, paravalvular regurgitation; TEE, transesophageal echocardiogram; ViV, valve‐in‐valve

TEE revealed a left ventricular ejection fraction of 55% to 60%, grade III diastolic dysfunction, and moderate PVR with two distinct jets in the noncoronary sinus (Figure 2A,B). The patient improved clinically with careful diuresis and further imaging with computed tomography suggested his aortic annulus actually measured 2.2 x 2.6 cm, larger than the 21 mm valve placed at surgery. Based on this, the PVR was felt to be due to the placement of an undersized 21 mm valve. Given excessive open surgical risk for this bedridden patient, transcatheter options were considered. This magna valve true inner diameter of 19 mm.^5^ ViV TAVR with balloon fracture of the surgical valve ring was felt the most appropriate method to address his regurgitation.^6^

A 23 mm Edwards Sapien 3 transcatheter valve was advanced “valve in valve” into the aortic annulus over a Confida wire with positioning confirmed by serial imaging and deployed under rapid pacing. Because the Magna valve (Edwards, Irvine, California) has a true inner diameter of 19 mm, the delivery system was subsequently exchanged over a wire for a 22 mm Atlas Gold Stiff Balloon (Bard Peripheral Vascular, Tempe, Arizona), which was used to post‐dilate with successful fracture of the prior surgical ring (Figure 2C).^5^ Intraoperative TEE revealed excellent functioning of the new valve with no aortic regurgitation and a mean gradient of 8 mmHg (Figure 2D). Postoperatively the patient improved remarkably and was walking at time of discharge to a rehab center.

## 4 DISCUSSION

Paravalvular leak following either TAVR or open surgical aortic valve replacement can be challenging and is best addressed by a multidisciplinary “Heart Team” approach with surgeons, interventional cardiologists, and cardiologists with imaging expertise.^7^ The two cases presented had multiple PVR jets with differing etiologies following TAVR and surgical aortic valve replacement. While both of these patients were deemed most suitable for ViV TAVR, an understanding of the disease process and additional technical options including occluder devices are paramount for comprehensive treatment plans.

### 4.1 Diagnosis

PVR after TAVR typically occurs due to malpositioning of the valve, which leads to a unfilled gap between the bioprosthesis and the native annulus.^8^ PVR is often related to annular calcification, improper sizing of the prosthesis to the native annulus, or underexpansion.^8^ Surgical replacement resulting in PVR can be related to tissue friability, annular calcification, or infection.^9^ Symptomatic patients most often present early after valve implant with symptoms of heart failure such as shortness of breath, left ventricular enlargement, and pulmonary edema.^9^, ^10^ Alternatively, a minority of patients can present with hemolysis from red cells traversing a high‐velocity narrow orifice resulting in symptomatic anemia.^9^, ^10^

Transthoracic echocardiography is used for initial imaging, but TEE is considered to be the diagnostic test of choice for PVR due to its superior sensitivity of 98% to 100% and specificity of 95% to 100% in localizing and grading the severity of the PVR.^2^ Grading of PVR after aortic valve replacement relies on both quantitative as well as qualitative measures. Complicating factors in this process include impairment of imaging from the valve stent frame coupled with native valve calcifications altering jet trajectories and shapes as well as acoustic shadowing that can require multiple imaging windows for adequate visualization.^11^ The following echocardiographic characteristics have been shown to be consistent with at least moderate aortic regurgitation: vena contracta width of >0.3 cm, vena contracta area > 0.1 cm^2^, a continuous circumferential area of regurgitation >10%, large flow convergence in the aorta, holodiastolic flow reversal in the abdominal aorta, regurgitant volume > 30 mL, regurgitant fraction >30%, or an effective regurgitant orifice area of >0.1 cm^2.^ ^11^ However, as identification and grading of PVR has many pitfalls, strong collaboration and communication between the cardiologists interpreting echocardiographic images and their interventional colleagues improves outcomes.^7^

### 4.2 Management

Medical management alone has not been shown to improve moderate or severe PVR.^2^ Treatment options include redo surgery, vascular occluder devices, ViV TAVR, or ViV TAVR with valve fracture of bioprosthetic surgical valves. There is a paucity of evidence related specifically to patients with multijet PVR. Our case series supports the assertion that ViV TAVR options are particularly suited for patients with larger crescentic PVR or multiple jet PVR as the etiology of such a complication is most likely due to undersizing of the previous valve. However, familiarity with multiple therapeutic options is vital for successful management as each case will have a unique set of circumstances and technical challenges.

Open surgical redo valve surgery for patients with asymptomatic bioprosthetic aortic regurgitation and acceptable surgical risk is a Class IIa recommendation.^12^ However, many patients with PVR are symptomatic on presentation or are higher risk surgical candidates which make redo sternotomy and open valve replacement less appealing^13^, ^14^, ^15^; furthermore, many patients do not want to undergo another major operation. Recent data has shown equivalent outcomes with transcatheter procedures compared to surgery even though the transcatheter patients had more comorbidities.^16^, ^17^, ^18^ In fact, adjusting for baseline characteristics, similar outcomes were observed between surgical and transcatheter procedures at 1 year.^13^, ^17^

**FIGURE 3:**
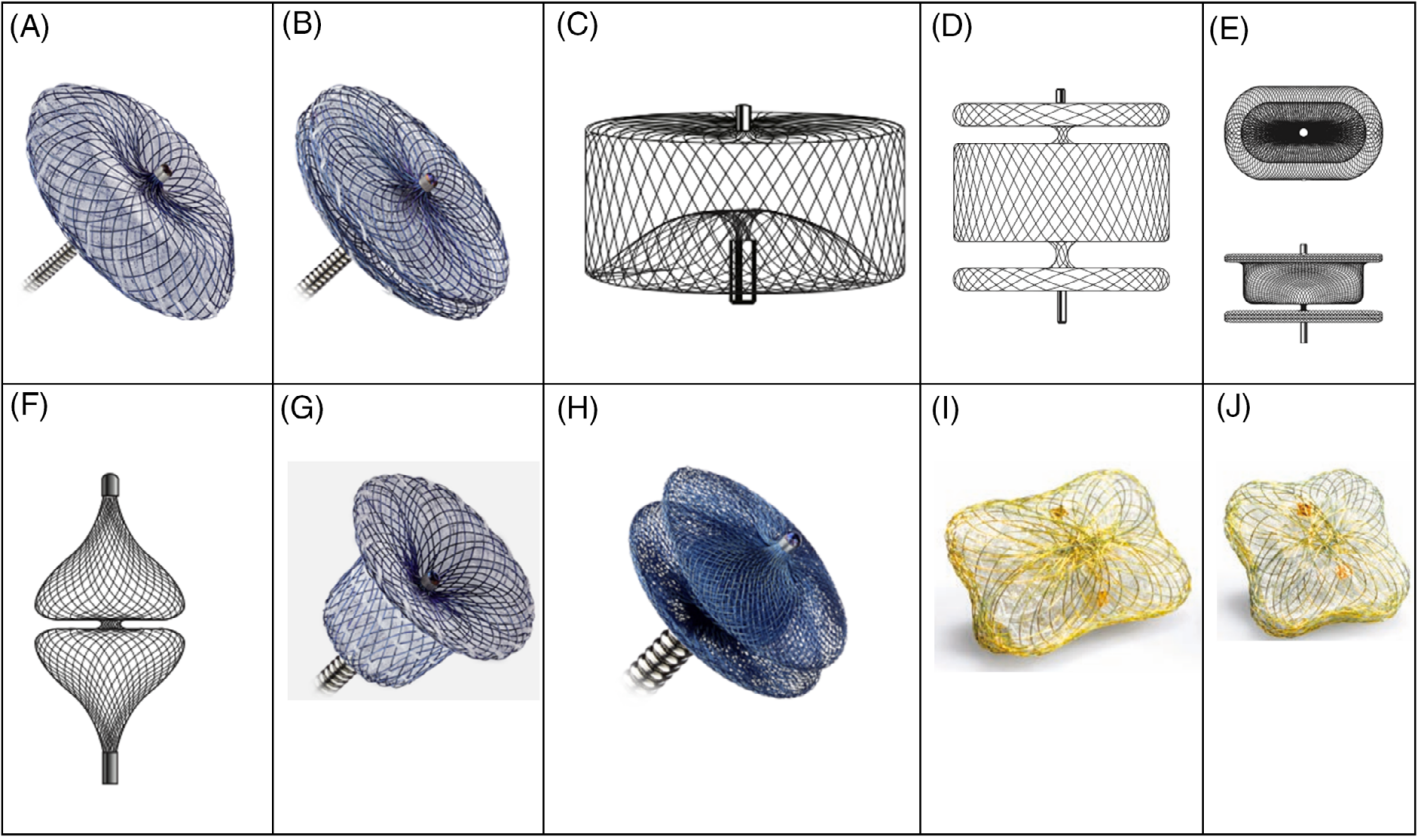
Vascular occluder devices. A, Amplatzer septal occluder^35^; B, Amplatzer cribriform multifenestrated septal occluder^35^; C, Amplatzer vascular plug^36^; D, Amplatzer vascular plug II^36^; E, Amplatzer vascular plug III^36^; F, Amplatzer vascular plug 4^36^; G, Amplatzer TM duct occluder^37^; H, Amplatzer TM duct occluder II^37^; I, Occlutech rectangular paravalvular leak device^38^; J, Occlutech square paravalvular leak device^38^

Accordingly, transcatheter treatment of PVR has emerged as an attractive modality. A Class IIa recommendation exists for percutaneous treatment of PVR in patients with lesser degrees of regurgitation but in whom New York Heart Association class III or IV symptoms exist, who have intractable hemolysis, or in whom improvement in hemodynamics is anticipated.^12^ While a number of devices have European approval, there are currently no occluder devices approved by the United States Food and Drug Administration specifically to treat PVR.^19^ The majority of data surrounding these self‐expanding occluder devices are from procedures treating a focal jet of PVR and have shown a high degree of success (>85%) and minimal rates (3.8%‐10%) of procedural complications.^9^, ^10^, ^13^, ^14^, ^20^, ^21^, ^22^ Devices available include the Amplatzer vascular occluders, Amplatzer duct occluders, Amplatzer septal occluders, and Occlutech Paravalvular Leak Devices, with selection of specific device tailored to the patient (Figure 3).^9^, ^18^, ^19^, ^23^, ^24^ Additional research to develop tailored devices is ongoing.^25^

**FIGURE 4:**
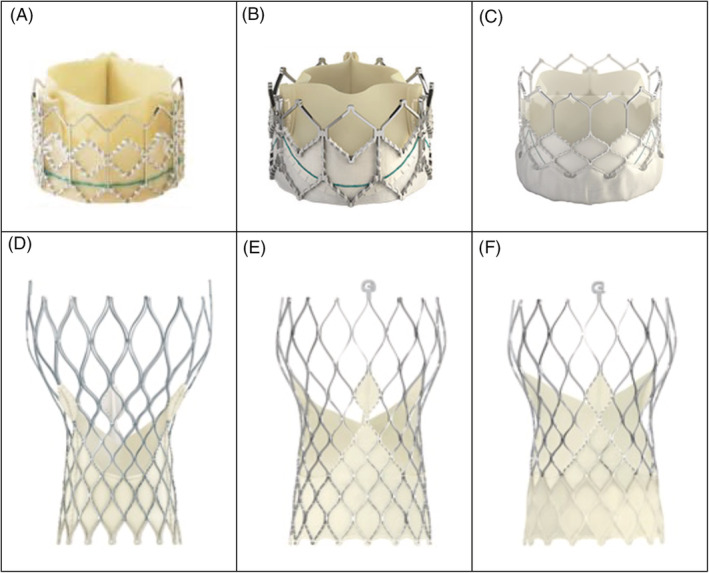
Comparison of TAVR valves. A, Edwards SAPIEN trancatheter heart valve^39^; B, Edwards SAPIENT XT transcatheter heart valve^40^; C, Edwards SAPIEN 3 transcatheter heart valve^41^; D, Medtronic CoreValve^42^ E, Medtronic corevalve evolut R^42^; F, Medtronic CoreValve Evolut Pro.^42^ TAVR, transcatheter aortic valve replacement

An alternative catheter‐based PVR treatment option is ViV TAVR. This procedure relies on bioprosthetic valves engineered for native valve pathology rather than for PVR. Furthermore, procedural success largely relies on operator expertise predominantly gathered through treatment of native valve aortic stenosis. However, data generated by this procedure has been favorable when compared to surgery.^16^, ^17^, ^26^ First generation TAVR valves reported moderate or severe PVR rates of 10.5% for the SAPIEN valve and 7.8% in the CoreValve. Second generation Sapien XT and Evolut R valves reported moderate or severe PVR at rates of 3.7% and 3.3% respectively.^27^, ^28^ However, current third generation valves continue to improve PVR rates with the SAPIEN 3 reporting a moderate or severe PVR rate of 0.5% and Evolut Pro reporting moderate or severe PVR in 2.9%.^1^, ^29^, ^30^ This improvement in PVR rates has largely been attributed to valve design—specifically the addition of an outer skirt at the ventricular inlet^3^ (Figure 4).

ViV techniques for PVR are limited to successful case reports demonstrating appropriate expansion of the newly implanted valves avoiding PVR.^31^, ^32^ A meta‐analysis did reveal higher rates of PVR with ViV TAVR compared to redo‐surgery (OR: 5.97, 95% CI: 1.40–25.44; *P* = .02); however, these data do not delineate the indication for such procedures and therefore may not be indicative of this specific sub‐population.^26^

Bioprosthetic valve fracture is an established technique used to expend the annulus of an undersized surgical valve. The largest series to date includes 75 patients with failed surgical valve replacements who have undergone bioprosthetic valve fracture with concomitant ViV TAVR. This study demonstrated a decrease in mean prosthetic valve gradient from 40.6 ± 15.8 to 8.1 ± 4.8 mm Hg (*P* < .001) with accompanying increase in mean effective orifice area from 0.8 ± 0.3 cm^2^ to 1.4 ± 0.8 cm^2^ (*P* < .001).^6^ Such results have important prognostic implications as decreased mean gradients across aortic valves are associated with improved mortality.^2^ These data also emphasize the importance of proper sizing at initial valve implantation and proper expansion if TAVR is initially employed. Overall, bioprosthetic valve fracture with ViV resulted in a PVR rate of 2.6% but notably was without aortic root disruption, coronary artery occlusion, or new pacemaker implantation.^6^ Within that cohort, there were three patients noted to have baseline PVR (graded trivial or mild) prior to bioprosthetic valve ring rupture, which resolved completely in all cases.^6^ Additional case reports also support treatment of PVR with ViV TAVR.^33^, ^34^

## 5 CONCLUSIONS

Treatment of PVR after bioprosthetic valve implantation requires knowledge of pathogenesis, diagnosis, and treatment of this disease process. While ideally avoided through appropriate valve sizing and technique, limited data exist regarding the optimal management strategy for patients with multiple jet PVR should it occur. The patients presented had undergone transcatheter and surgical aortic valve replacements and both benefitted from ViV technology. Adjunctive maneuvers including the use of an Amplatzer Duct Occluder in one case and bioprosthetic valve fracture in the other ensured optimal outcomes. With the knowledge that moderate or severe PVR is associated with increased mortality, these cases highlight successful treatment strategies for multiple jet PVR as well as the importance of familiarity with multiple therapeutic options to achieve optimal results.

## CONFLICT OF INTEREST

The authors declare that they have no conflicts of interest.

## ETHICS STATEMENT

This article does not contain any studies with human participants or animals performed by any of the authors.

## Supporting information


**Appendix** S1: Supporting information
